# *Scopolia carniolica var. hladnikiana*: Alkaloidal Analysis and Potential Taxonomical Implications

**DOI:** 10.3390/plants10081643

**Published:** 2021-08-10

**Authors:** Karsten Fatur, Matjaž Ravnikar, Samo Kreft

**Affiliations:** Department of Pharmaceutical Biology, Faculty of Pharmacy, University of Ljubljana, 32 Tržaška cesta, 1000 Ljubljana, Slovenia; Matjaz.ravnikar@ffa.uni-lj.si (M.R.); samo.kreft@ffa.uni-lj.si (S.K.)

**Keywords:** *Scopolia carniolica*, *Scopolia carniolica var. hladnikiana*, taxonomy, secondary metabolites, tropane alkaloids, Slovenia, Solanaceae

## Abstract

The present research sought to compare the content of hyoscyamine/atropine and scopolamine in *Scopolia carniolica* and its contested variety, *S. carniolica var. hladnikiana*, with the aim of investigating differences that may be of taxonomical significance. A multi-phase liquid extraction and high-performance liquid chromatography were used to extract and analyse these alkaloids in different organs from plants collected over two years at three sites. Our results showed that hyoscyamine was almost twice as prevalent as scopolamine across our 87 samples. The differences between organ types were large, but so too were intra-organ differences; differences due to organs proved to be significant for hyoscyamine, while they were only marginally significant for scopolamine. The collection site also proved to have a significant influence, but only on hyoscyamine content. The year of collection and the variety proved to not be significant. Our results support the theory that these two varieties are likely one, a view argued by many others, though more work is needed to draw concrete taxonomical conclusions.

## 1. Introduction

Named after the famed botanist, Franc Hladnik, who first discovered it, *Scopolia carniolica var. hladnikiana*, otherwise known as Hladnik’s *Scopolia* is a plant from the Solanaceae family that is endemic to a small number of discontinuous sites throughout the south eastern European country of Slovenia [1,2]. While the common *S. carniolica* has a distribution spanning throughout central and southeast Europe, the Hladnik variety (as of 2013) grows in only 10 confirmed sites [3]. Both varieties tend to grow in ravines within deciduous forests, especially those with high numbers of *Fagus* spp. [4,5]. The Hladnik variety is found growing in the presence of the normal variety.

These glabrous plants are small, deciduous perennials, reaching about 20–60 cm in height and appearing in early spring [4]. Growing up from the rhizomatic rootstock, the simple, ovate leaves (~10–20 × 5–8 cm) are found on short petioles alternately arranged on the stem and have simple to slightly crenate margins [4]. Campanulate flowers (15–25 mm) with floral parts of five, bloom in early spring from the leaf axils on comparably long pedicels and mature into capsules [4].

The primary distinction between *S. carniolica* and the Hladnik variety is that of the flower colour; while the standard variety has flowers with a deep violet colour on the outside of the corolla and a yellow colour on the inside, the Hladnik variety is a yellow-green throughout [2,4]. That being said, many other differences have been stated at various points in time, such as the Hladnik variety having shorter leaves, larger flowers, a later flowering time, and fewer flowers, while the regular variety is said to have purple leaf veins that are absent in the Hladnik variety [2,4]. However, these differences have largely proven to be inconsistent, with flower colour being the primary means of differentiating. It is now debated by many whether the Hladnik version is in fact a true variety or if it falls within the normal variation for *S. carniolica*.

Where previous investigations into the divide between *S. carniolica* and the Hladnik variety were morphologically focused, a comparative analysis of alkaloids has yet to be performed to see if there are any clear differences in the chemical makeup of these two plants.

Along with other close relatives in the Solanaceae, *S. carniolica* contains the tropane alkaloids scopolamine and hyoscyamine (which racemises to atropine when dried), which found extensive use throughout the years as a result of their multitude of effects in the human body [6,7,8]. Synthesised in the roots and then spread throughout the other organs, these alkaloids serve to protect the plants from predation as well as from various other forms of life and pathogens [9,10].

This research sought to examine the levels of the alkaloids hyoscyamine/atropine and scopolamine in samples of both *S. carniolica* and *S. carnolica var. hladnikiana*, comparing variety, organ, year of collection, and collection site to determine if there is any significant difference in alkaloid levels between the two plants that would support their division into separate varieties.

## 2. Results and Discussion

Through our analysis of standards of atropine and scopolamine and our re-extraction of certain samples, our method was validated and confirmed by its ability to extract and analyse these alkaloids from solution or plant material. The recovery rate for standard extraction was higher than 78% for atropine and 95% for scopolamine, while the efficacy of the extraction was higher than 83% for atropine and 87% for scopolamine; the samples had a relative standard deviation of 2.7%.

On average, the content of scopolamine in our samples was 692 µg/g (=0.07%), while the content of atropine was approximately double at 1379 µg/g (=0.14%). This higher content of hyoscyamine/atropine than scopolamine is consistent with research on other closely related plants [11,12,13,14,15,16]. That being said, the content of these alkaloids changes drastically between growth phases in related plants and may do so in *S. carniolica* as well, though hyoscyamine/atropine is generally predominant [17].

As displayed in Table 1, the content of both alkaloids was greatly varied between the different organs. However, the differences even within the organ groups are large; as a result, the ANOVA test for the differences of scopolamine between the organs is only marginally significant (*p* = 0.059). The differences for atropine, however, are significant (ANOVA *p* = 0.006). Previous research in Slovenia has likewise shown a great deal of variation between *S. carniolica* plants in their content of atropine and scopolamine [18], a trend that was also observed in closely related plants with the same alkaloids [10].

Our research exhibited the highest content of scopolamine in the leaves and lowest in the roots, while the content of hyoscyamine/atropine was highest in the roots and lowest in the flowers. The previously mentioned prior research in Slovenia completely matched this pattern, except for finding that the highest quantity of scopolamine was in the flowers [18]. As *S. carniolica* and its close relatives are believed to synthesise hyoscyamine/atropine in their roots, its high concentration there is unsurprising; similarly, as the flowers are the most ephemeral part of the plant and most removed from the roots, it is reasonable that this alkaloid would be least present there [10]. Hyoscyamine/atropine is also the precursor for scopolamine, a change that is catalysed by hyoscyamine-hydroxylase (H6H); the higher content of scopolamine in the leaves rather than the roots may suggest that this process is largely carried out after the alkaloids leave the roots [10,19,20,21]. Further research with higher significance values for the scopolamine differences is needed to confirm this.

The average content of each alkaloid was highest from the Bohor mountain collection site (see Table 2), but only the differences for atropine were statistically significant (ANOVA *p* = 0.017). The differences in scopolamine and the total alkaloid content were not significant (*p* = 0.689 and *p* = 0.099 respectively). Seeing such a variation between sites was in line with our predictions, as previous research showed a range of environmental factors such as soil nitrogen, water stress, and pest type and number can affect the levels of these alkaloids found in plants [22,23,24,25].

We found no significant differences in the alkaloid content between different varieties/types of *Scopolia carniolica* (see Table 3). ANOVA *p* values were 0.800, 0.399, and 0.844 for scopolamine, atropine, and total alkaloids, respectively. Previous research with related plants showed demonstrably different alkaloidal levels between varieties grown in the same conditions, so this is either not a trait seen in *S. carniolica*, or the effect is concealed by other (random or environmental) effects, or the two varieties are not distinct [26,27].

Indeed, not only was the alkaloid difference not significant, but the reported morphological differences that are so clearly laid out in the literature did not present themselves at all. The supposedly clear-cut distinction between the yellow-green flowers of the Hladnik version and the purple outside and yellow inside of the main variety was a nebulous continuum between the two [28]. Even referring to it as a continuum, however, is generous; though many flowers were intermediary in their colours, we also collected an individual with flowers in stripes of purple and yellow running the length of the corolla.

As previously stated, several other differences were noted with lesser frequency by various authors, such as the Hladnik variety having shorter leaves, larger and fewer flowers, and a later flowering time. These patterns were not observed in our collections, and in fact the opposite was equally as likely to be true, making it seem that these were all within the normal variability of the species. Similarly, the presence of purple veins was meant to be present in only one variety but was observed in plants with a range of flower colours.

We found no significant differences in the alkaloid content between different years of collection (see Table 4). Though many environmental conditions can shift and affect alkaloid production within a site on a given year, it is clear that the two subsequent years sampled for this research were similar enough as to not significantly impact this process.

To further analyse the data, we created four general linear models (Table 5), two for each alkaloid. In one model we assumed that independent variables (organ, location, type, and year) influence the dependant variables (scopolamine and atropine content) without interaction. In the other model, we assumed there could be all possible two-way interactions. The results were similar to our ANOVA analysis: atropine content is highly influenced by organ type, which displays a weaker yet present influence on scopolamine content. Location also proved to influence alkaloid content. The year of collection and the variety of the plant, however, did not significantly impact the quantity of alkaloids in the plants.

**Figure 1:**
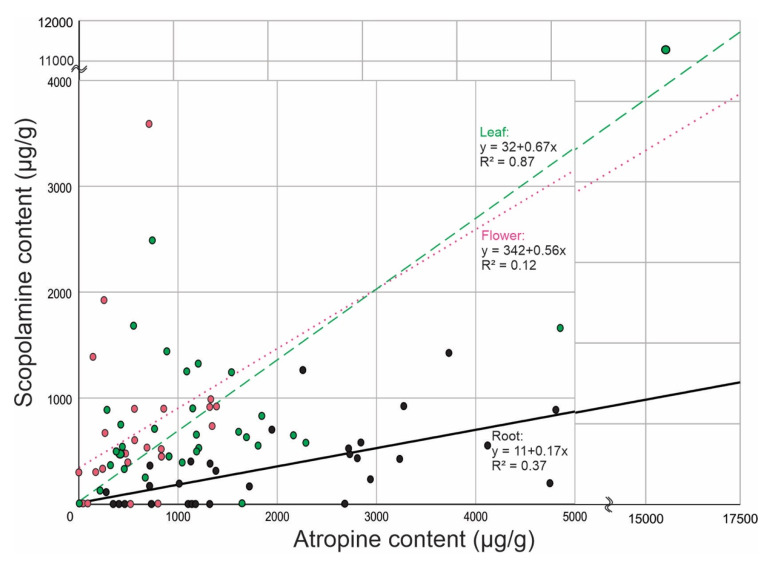
Grouped scatter plot of scopolamine and atropine by organ.

As exhibited in Figure 1, the content of one alkaloid correlated with the content of the other (*p* = 0.000001 for all samples and 0.071, 0.000001 and 0.000317 for flower, leaf and root respectively).

**Figure 2:**
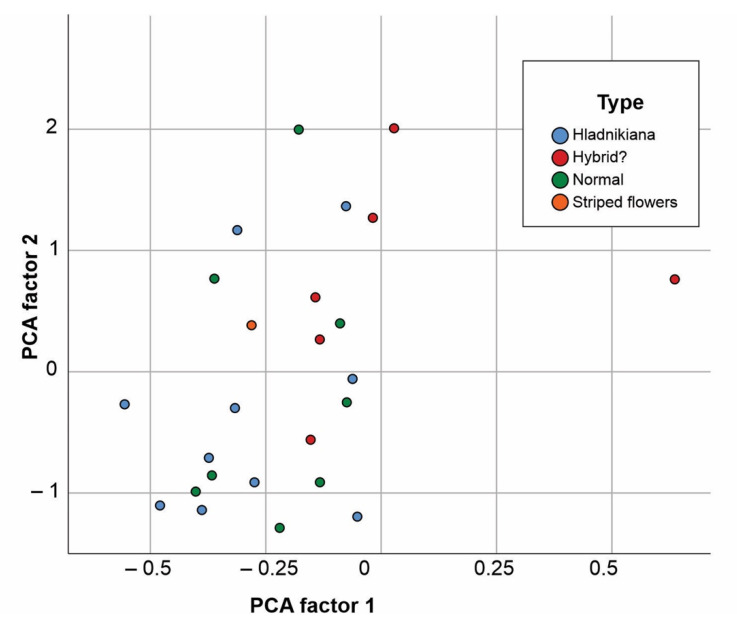
PCA analysis with six dependent variables (atropine or scopolamine by leaf, root and flower) in individual specimens.

We performed a PCA analysis, where six dependent variables (atropine or scopolamine by leaf, root and flower) in individual specimens were used. The results of the PCA (see Figure 2) reinforce the observation about the lack of classification of visual phenotypes by phytochemical variables.

## 3. Materials and Methods

### 3.1. Sample Collection

Samples were collected during flowering, with a collection period of 1–9 April 2019 and 24 March–4 April 2020. A total of 31 plants were sampled (8 of the common variety, 15 Hladnik’s variety, and 8 that morphologically fell between the two, all classified by corolla colour) from three different locations: the Ljubljana botanical garden (eight plants in 2019 and six plants in 2020), Pekel pri Borovnici (three plants in 2019), and Bohor mountain (six plants in 2019 and seven plants in 2020). Not all of the plants had flowers.

### 3.2. Sample Preparation

Plants were divided into roots, flowers (including the pedicels), and leaves/stems. Most had all three organ types available, but this was not always the case as some did not have enough flowers for analysis; as a result, we had a total of 87 samples rather than 93. Samples were then dried at room temperature and ground.

### 3.3. Extraction

The methods used for extraction and analysis were based upon previously published research [29] with minor alterations.

A total of 0.25 g of the dried plant matter was weighed out and then transferred into a 50 mL beaker by rinsing with 3 × 5 mL methanol. The beaker was then covered with parafilm into which small holes were poked and then put into an ultrasonic bath without additional heat for 1 h.

The extract was then filtered into a 50 mL round bottom flask using round format cellulose qualitative filter paper MN 615 Grade (Macherey-Nagel, Duren, Germany). A total of 5 mL of methanol was used to rinse the beaker and filter into the flask afterwards. The ethanolic extract was then completely evaporated in a rotary evaporator with a 40 °C water bath at 200 mbar.

The remaining dry extract was resuspended with 25 mL of 0.5% HCl and then poured into a 250 mL separatory funnel. A total of 20 mL of dichloromethane was then used to rinse the flask into the separatory funnel as well. The funnel was then capped and shaken to carry out liquid-liquid extraction. The mixture was allowed to sit until separation of the phases had occurred, and the lower layer (dichloromethane) was then removed and discarded. Two more rounds of 20 mL dichloromethane were repeated in this manner.

Then 1 mL of 30–33% ammonium hydroxide solution was added to the remaining (water) phase to raise the pH to 10. The sample was then shaken to ensure proper mixing.

A total of 20 mL of dichloromethane were added and extracted as previously, with the only difference being that the dichloromethane was collected and kept. This process was repeated with an additional four rounds of 20 mL of dichloromethane, collecting all dichloromethane extracts into the same 250 mL beaker. The remainder (water phase) in the separatory funnel was discarded after completion of this step.

Then 15 g of anhydrous sodium sulphate was added to the dichloromethane extract and mixed. This was then filtered into a 100 mL round bottom flask, with an additional 2 mL of dichloromethane being used to rinse the beaker and filter.

This solution was evaporated in a rotary evaporator with a 40 °C water bath at 700 mbar. Once evaporated, 2 × 2.5 mL of methanol were used to dissolve the dry extract and transfer it into a test tube. For samples from which there was too little to attain 0.25 g of dried plant matter at the beginning of extraction (primarily the flower samples), a corresponding amount of methanol was used in this step to ensure equal concentration of the alkaloids.

Standards of atropine (Sigma, St. Louis, MO, USA) and scopolamine hydrochloride (Fluka, Charlotte, NC, USA) were also extracted using the same extraction process to ensure viability of the method; sample re-extraction was also performed to determine effectiveness.

Samples were then filtered for HPLC and additional volume was stored in case of further need.

### 3.4. Analysis

For identification via reference standards and quantification of the compounds, the HPLC system (Shimadzu Prominence) was used. It consisted of a system controller (CBM20A), a column oven (CPO-20AC), and a solvent delivery pump with a degasser (DGU20A5) connected to a photodiode array detector (SPD-M20A) that monitored wavelengths 190–800 nm. The responses of the detectors were recorded using LabSolution software version 5.71. The chromatography was performed at 40 °C and a flow rate of 2 mL min, using a Phenomenex Kinetex^®^ C18 column (10 cm × 4.6 mm I.D., 2.7 µm particle size). We utilised the following gradient method with solvent A (water with 2% acetonitrile and 0.1% trifluoroacetic acid) and solvent B (acetonitrile with 2% water and 0.1% trifluoroacetic acid): 0–1 min 3% B, 1–10 min 3–20% B, 10–12 min 20–100% B and 12–15 min 100–5% B. Samples and standards were detected at an absorbance of 210 nm and were injected into the HPLC system at a volume of 10 µL.

## 4. Conclusions

The debate on the status of *Scopolia carniolica var. Hladnikiana* as a separate variety from the main species is old, and unlikely to be settled simply through the present research. In combining our results with established literature to compare, it seems more likely that these morphological differences are a simple variation in the species rather than being attributable to varietal labels.

As the first published chemical comparison of these supposed varieties, we observed that there is in fact no significant connection between the morphological features and the content of its main alkaloids, hyoscyamine/atropine and scopolamine. Though this in itself does not prove that these plants should all be considered without a varietal divide, it does seem suggestive of such conclusions. The botanical observations noted during collection further point in this direction; however, molecular work is needed on this topic to confirm the level of difference between these organisms.

At this point it is worth noting that the most consistent variation between the two supposed types was seen at the Ljubljana Botanical Garden site; indeed, this is where the type collection for *S. carniolica var. Hladnikiana* that was found by Franc Hladnik was planted and still grows. Perhaps Hladnik’s individual samples are indeed representative of a distinct variety (perhaps his detractors would even go so far as to suggest he selectively bred the plant in private to amplify its features before announcing it publicly) or perhaps it is just another individual within the normal distribution of the species upon which a varietal type was arbitrarily constructed. Future research using quantitative measurements to examine the sizing of various morphological traits and studies able to quantify the colour of the flowers is needed to reach further conclusions; similarly, future research should focus on the chemical composition of these plants in terms of other substances besides the alkaloids here investigated, to see if the chemical fingerprint of this plant holds any secrets to its taxonomic troubles.

## Figures and Tables

**Table 1:** The content of scopolamine and atropine (µg/g) in different organs of *S. carniolica* (summarised for all locations, varieties, and years).

		Flower	Leaf	Root	Total
Scopolamine	Mean	619	1103	346	692
	N	28	31	31	90
	SD	748	1964	379	1273
Atropine	Mean	492	1615	1945	1379
	N	28	31	31	90
	SD	460	2737	1328	1887
Total alkaloids (Scopolamine + atropine)	Mean	1110	2717	2291	2070
	N	28	31	31	90
	SD	1004	4621	1585	2967

**Table 2:** The content of scopolamine and atropine (µg/g) in different locations of *S. carniolica* (summarised for all organs, varieties, and years). The ANOVA *p* is 0.689 for scopolamine and 0.017 for atropine.

		Bohor Mountain	Ljubljana Botanical Garden	Pekel Pri Borovnici
Scopolamine	Mean	816	585	570
	N	28	31	31
	SD	1695	794	381
Atropine	Mean	1942	758	1445
	N	28	31	31
	SD	2428	965	1204

**Table 3:** The content of scopolamine and atropine (µg/g) in different varieties/types of *S. carniolica* (summarised for all organs, locations, and years). The ANOVA *p* is 0.800 for scopolamine and 0.399 for atropine.

		Hladnikiana	Hybrid?	Normal	Striped Flowers
Scopolamine	Mean	617	638	906	386
	N	28	31	31	3
	SD	1748	388	863	50
Atropine	Mean	1313	1934	987	1363
	N	28	31	31	3
	SD	2446	1269	1137	1313

**Table 4:** The content of scopolamine and atropine (µg/g) in *S. carniolica* collected in different years (summarized for all organs, locations, and varieties). The ANOVA *p* is 0.799 for scopolamine, 0.724 for atropine, and 0.908 for total alkaloids.

		2019	2020
Scopolamine	Mean	659	728
	N	28	31
	SD	1613	730
Atropine	Mean	1446	1304
	N	28	31
	SD	2357	1163

**Table 5:** The results of general linear model.

		Scopolamine		Atropine	
Source of variability	df	Sig.	Sig.	Sig.	Sig.
Corrected model	23	0.391	0.201	0.167	0.014
Intercept	1	0.000	0.007	0.000	0.000
organ	2	0.744	0.054	0.095	0.006
Location	2	0.134	0.124	0.083	0.051
Type	3	0.235	0.211	0.981	0.985
year	1	0.629	0.949	0.541	0.375
Location × type	0	na		na	
organ × location	4	0.452		0.380	
Location × year	1	0.130		0.459	
organ × type	6	0.683		0.657	
Type × year	2	0.373		0.367	
organ × year	2	0.328		0.503	
Error	66				
Total	90				
Corrected total	89				

## Data Availability

All data is reported within the manuscript.
